# Supplementary material for: The Flemmingsome reveals an ESCRT-to-membrane coupling via ALIX/syntenin/syndecan-4 required for completion of cytokinesis
Source: Nat Commun. 2020 Apr 22;11:1941. doi: 10.1038/s41467-020-15205-z (PMC7176721; doi:10.1038/s41467-020-15205-z)
Supplement: Supplementary file 1 — Supplementary Information [file 41467_2020_15205_MOESM1_ESM.pdf]

Supplementary Information

The Flemmingsome reveals an ESCRT-to-membrane coupling

via ALIX/syntenin/syndecan-4 required for completion of cytokinesis

Addi et al.

7 Supplementary Figures

1 Supplementary Table

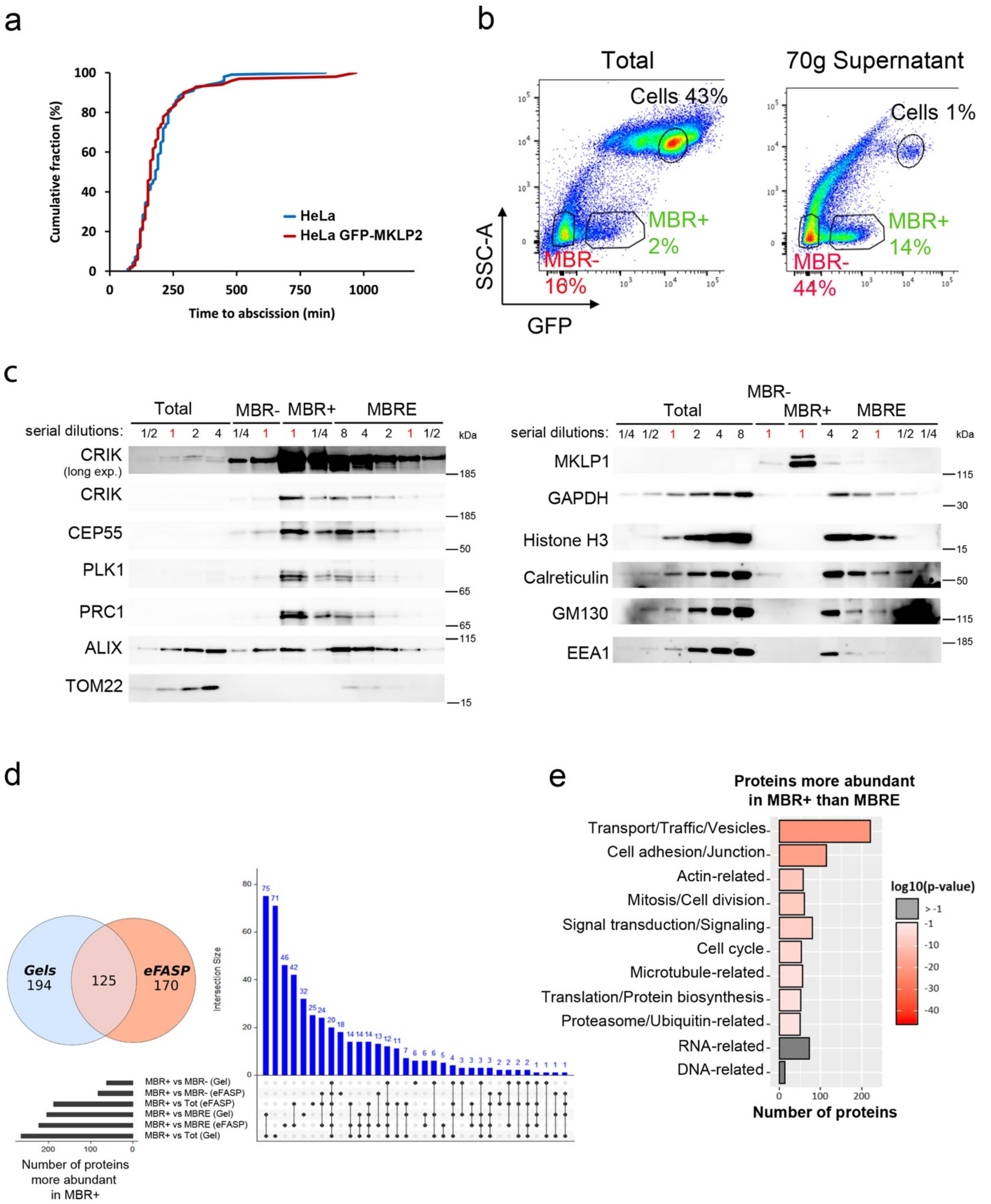

Supplementary Figure 1

Supplementary Fig. 1: Isolation and characterization of MBRs from GFP-MKLP2 cells.

(a) Abscission time was determined by phase-contrast time-lapse microscopy (recorded every 10 min). Cumulative plots of the abscission times in HeLa and in the HeLa-GFP-MKLP2 cell line used for proteomic analysis are presented. n=99 cells per cell line, no significant difference was found between the two cell lines (KS test, p= 0.136)

(b) Isolation of MBRs by flow cytometry. *Left panel.* GFP-MKLP2 cells were treated with EDTA (to detach cells from the substrate and MBRs from cell surface) (Total) and analyzed by flow cytometry (see also Fig. 1a). Gates show 43% Cells and distinct populations of small GFP-positive MBRs (2% MBR+) and GFP-negative particles (16% MBR-) in the low SSC population. *Right panel.* For comparison: supernatant from previous condition after a 70g centrifugation step, leading to cell depletion (1% remaining cells) and enrichment of MBR+ (14%) and MBR- (44%) populations (same graph as in Fig. 1b). The 70g supernatant was used for the sorting of MBR+ and MBR- fractions for the proteomic analysis. Several independent flow cytometry sortings were pooled and MBR+ and MBR- particles concentrated at 1200g to obtain enough MBR+ and MBR- protein extracts for the proteomic analysis.

(c) Uncropped Western blots of Fig. 1c, with indicated serial dilutions of protein extracts from total (Tot), Midbody-Enriched (MBRE), flow cytometry-sorted MBR- and MBR+ fractions. The same membrane was blotted repeatedly with indicated antibodies. Sypro-Ruby staining was used to calibrate the protein quantity before membrane blotting with the indicated antibodies (same amount of proteins in lanes labeled as “1”).

(d) UpSet plot and Venn diagram showing the significantly more abundant proteins in the MBR+ than in the 3 different controls (MBR-, MBRE, Total) with 2 different sample preparation techniques (eFASP or Gels). When merging all the results, 489 proteins were found significantly enriched in at least one of the comparisons: 194 are found uniquely enriched using Gels and 170 uniquely found when using eFASP while 125 are found enriched with both techniques.

(e) Over-representation analysis of GO term clusters for the proteins found statistically more abundant in MBR+ than MBRE. The size of each bar corresponds to the number of proteins associated with each cluster and the red gradient is function of the enrichment p-values coming from hypergeometric tests. Grey color means p-value >0.1.

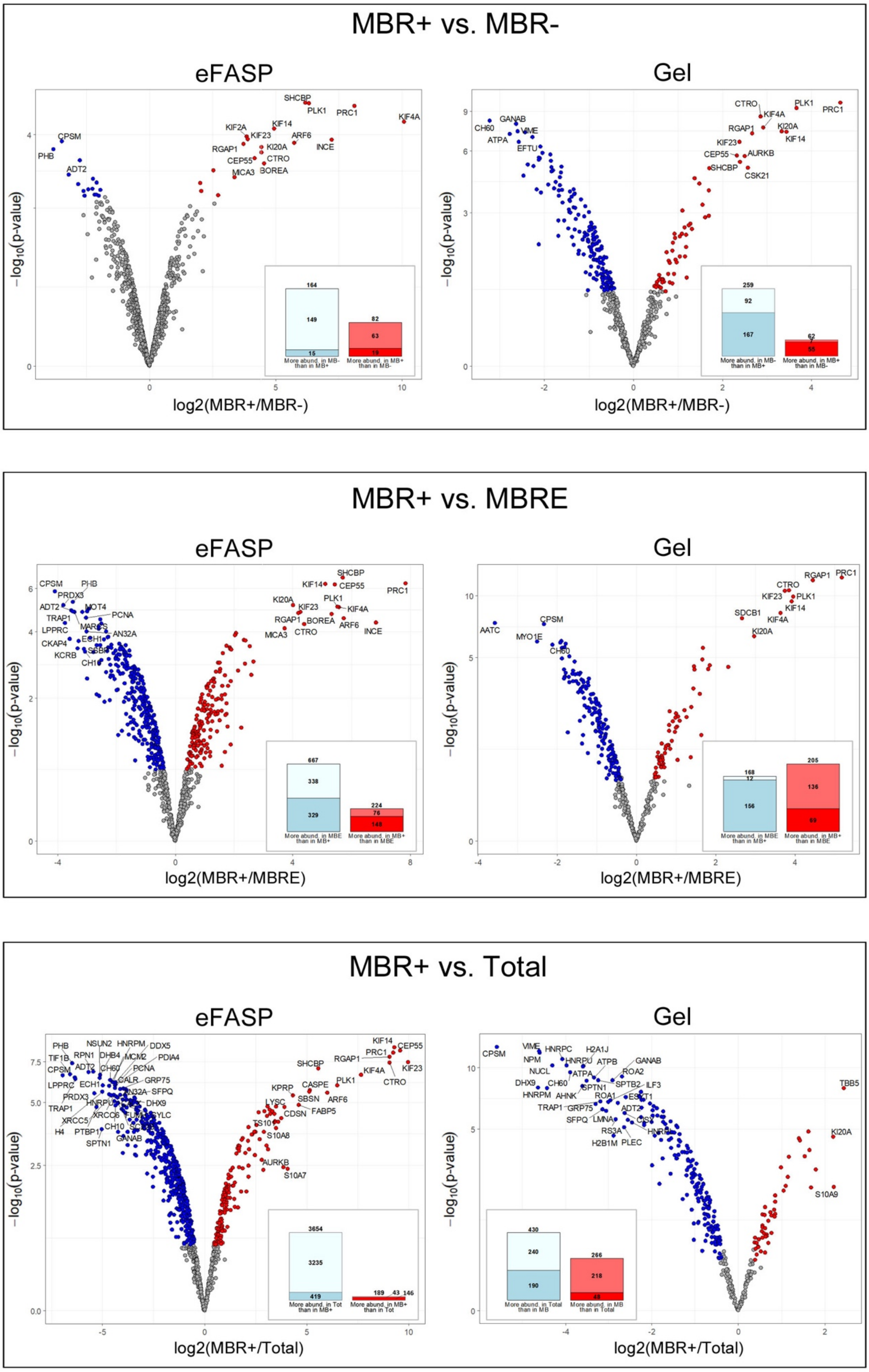

Supplementary Fig. 2

Supplementary Fig. 2: Characterization of the *Enriched Flemmingsome* using comparisons between proteomes of MBR+ vs. other fractions.

Volcano plots showing the proteins that are significantly more abundant in MBR+ or in the different used controls [MBR-, MBRE or Total] with either eFASP or Gels and quantified in both conditions. The proteins that are significantly more abundant in MBR+ are represented in red and the ones significantly more abundant in controls are in blue. The x-axis represents the log2 ratio between average intensities in MBR+ and average intensities in controls and the y-axis is the -log10(p-value) where the p-value has been computed with a LIMMA t-test. Bar plots represent the total number of proteins that are more abundant in MBR+ than in the different controls (proteins of volcano plot + proteins only quantified in a condition) and vice versa (light blue: proteins only quantified in the control, blue: proteins significantly more abundant in the control and selected by the statistical test, light red: proteins only quantified in MBR+, red: proteins significantly more abundant in MBR+ and selected by the statistical test).

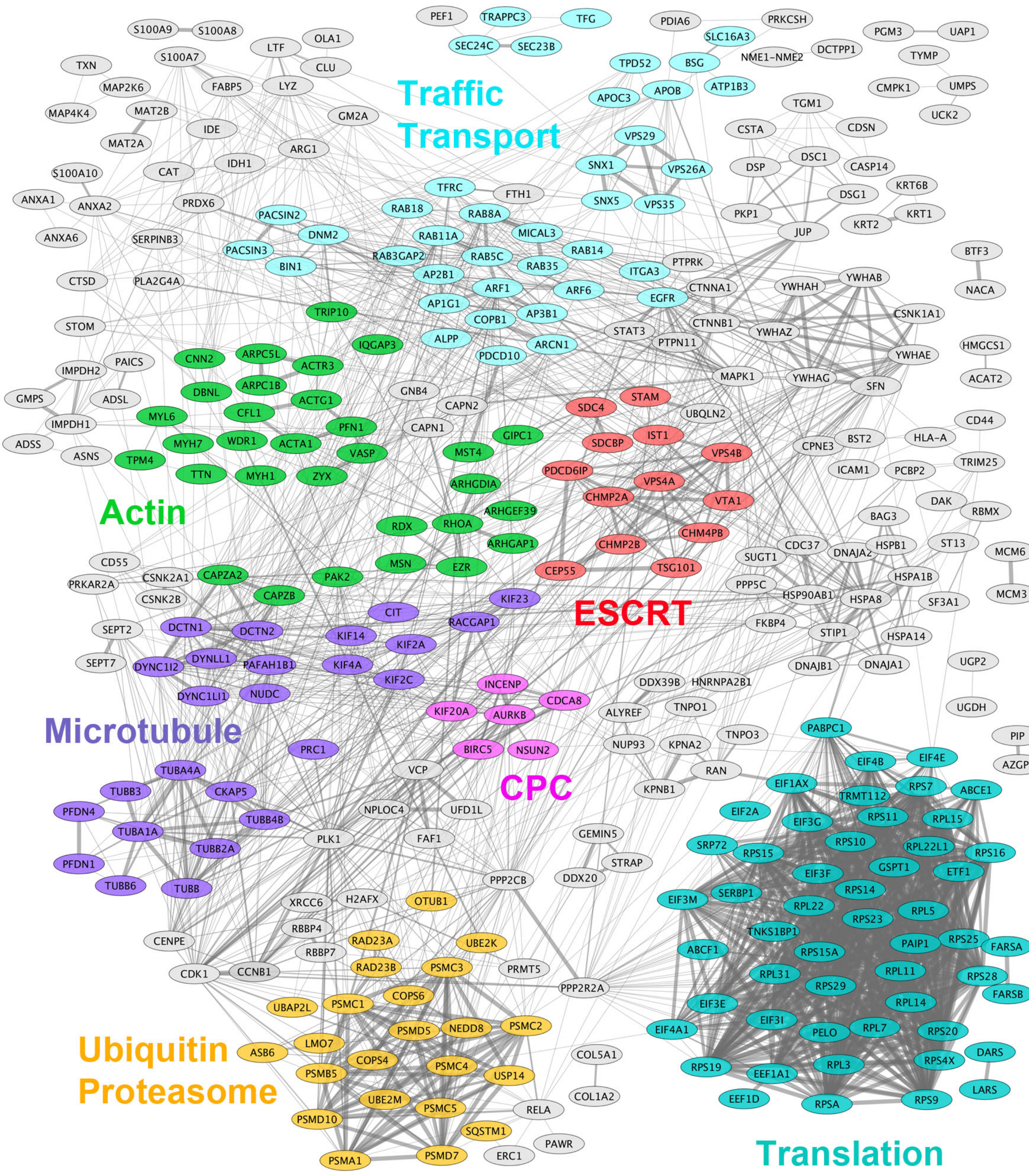

Supplementary Fig. 3

Supplementary Fig. 3: STRING functional association network for the *Enriched Flemmingsome* (Zoom on the STRING association network displayed in Fig. 1f.).

Proteins with known interactions (337 of 489) are displayed (see also Supplementary Table 1, TAB2). Based on manually-curated literature search, colours group proteins from same functional categories, as indicated, using Cytoscape. Widths of the edges correspond

to the *combined score* of STRING reflecting the confidence placed in each interaction. Only phylogenetic co-occurrence interactions, experimentally determined interactions and database annotated interactions have been considered. CPC: Chromosomal Passenger Complex-related.

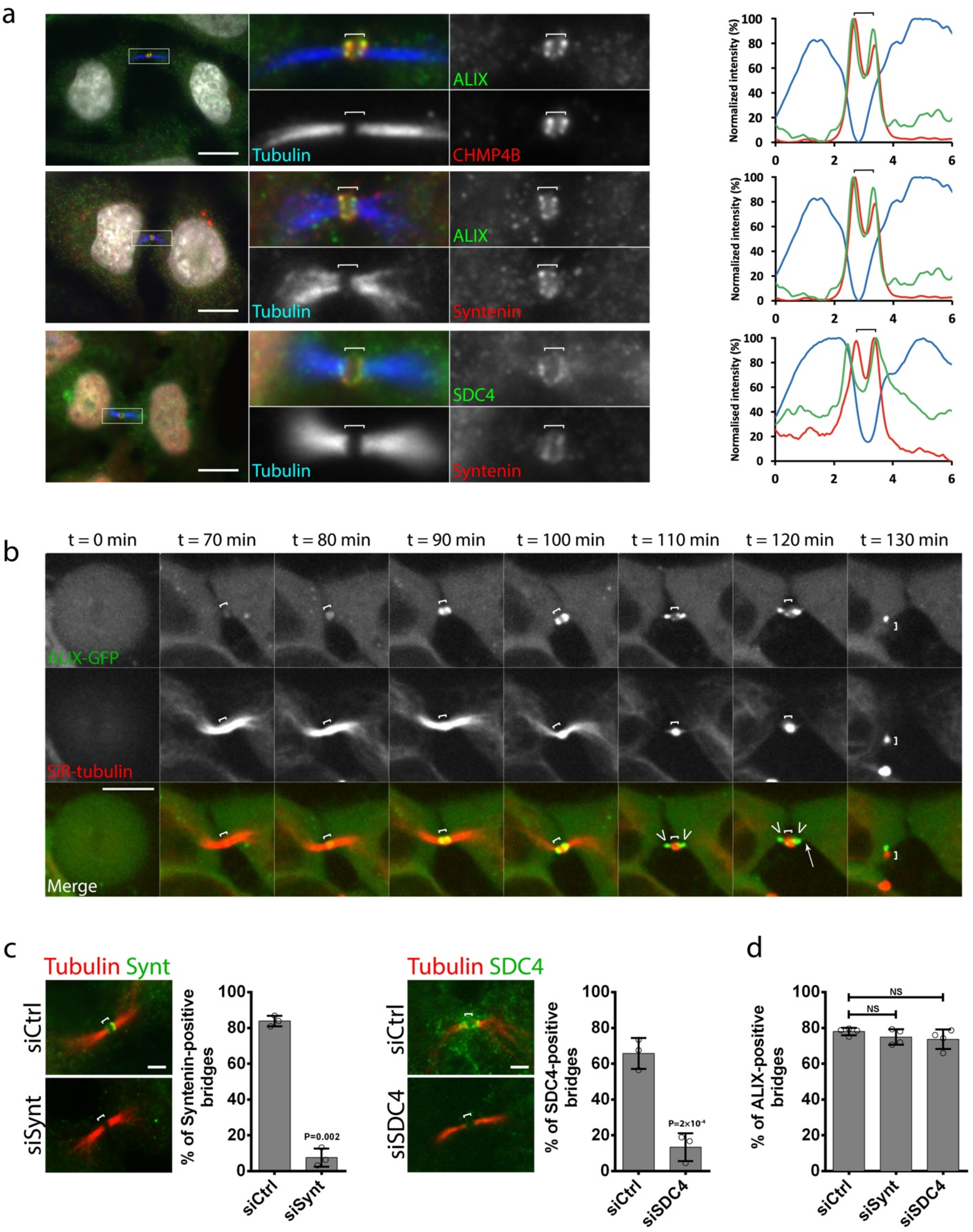

Supplementary Figure 4

Supplementary Fig. 4: Localization of syntenin, syndecan-4 and ALIX in wild-type and depleted cells.

(a) *Left panels:* Endogenous localization of ALIX, CHMP4B, syntenin, syndecan-4 (SDC4) and acetylated-tubulin in late bridges without abscission site in fixed HeLa cells, as indicated. We used an antibody recognizing the ectodomain of SDC4 for the

immunofluorescence. *Right panels:* Intensity profiles along the bridge of the corresponding images with matched colours from left panels. Scale bar: 10 µm.

(b) HeLa cells transiently transfected with a plasmid encoding ALIX-GFP and incubated with fluorescent SiR-tubulin were recorded by spinning-disk confocal microscopy every 10 min. Time 0 corresponds to the time frame preceding furrow ingression. Scale bar: 10 µm.

(c) Specificity of antibody stainings. *Left panel:* late cytokinetic bridges stained for endogenous syntenin and acetylated-tubulin, in control and syntenin siRNAs-treated cells, as indicated. Percentage of bridges positive for syntenin in each condition. n=31-53 cells, N=3 independent experiments. *Right panel*: late cytokinetic bridges stained for endogenous syndecan-4 (SDC4) and acetylated-tubulin, in control and syndecan-4 siRNAs-treated cells, as indicated. Percentage (mean ± SD) of bridges positive for syntenin in each condition. n=22-37 cells, N=3 independent experiments, one-sided Student's t-tests. Scale bar : 2 µm.

(d) Quantification of the proportion of bridges positive for ALIX in control, syntenin and syndecan-4 siRNAs-treated cells. n=25-64 cells, N=4 independent experiments, one-sided Student's t-tests.

NS: non significant.

Brackets and arrowheads mark the midbody and the abscission site, respectively.

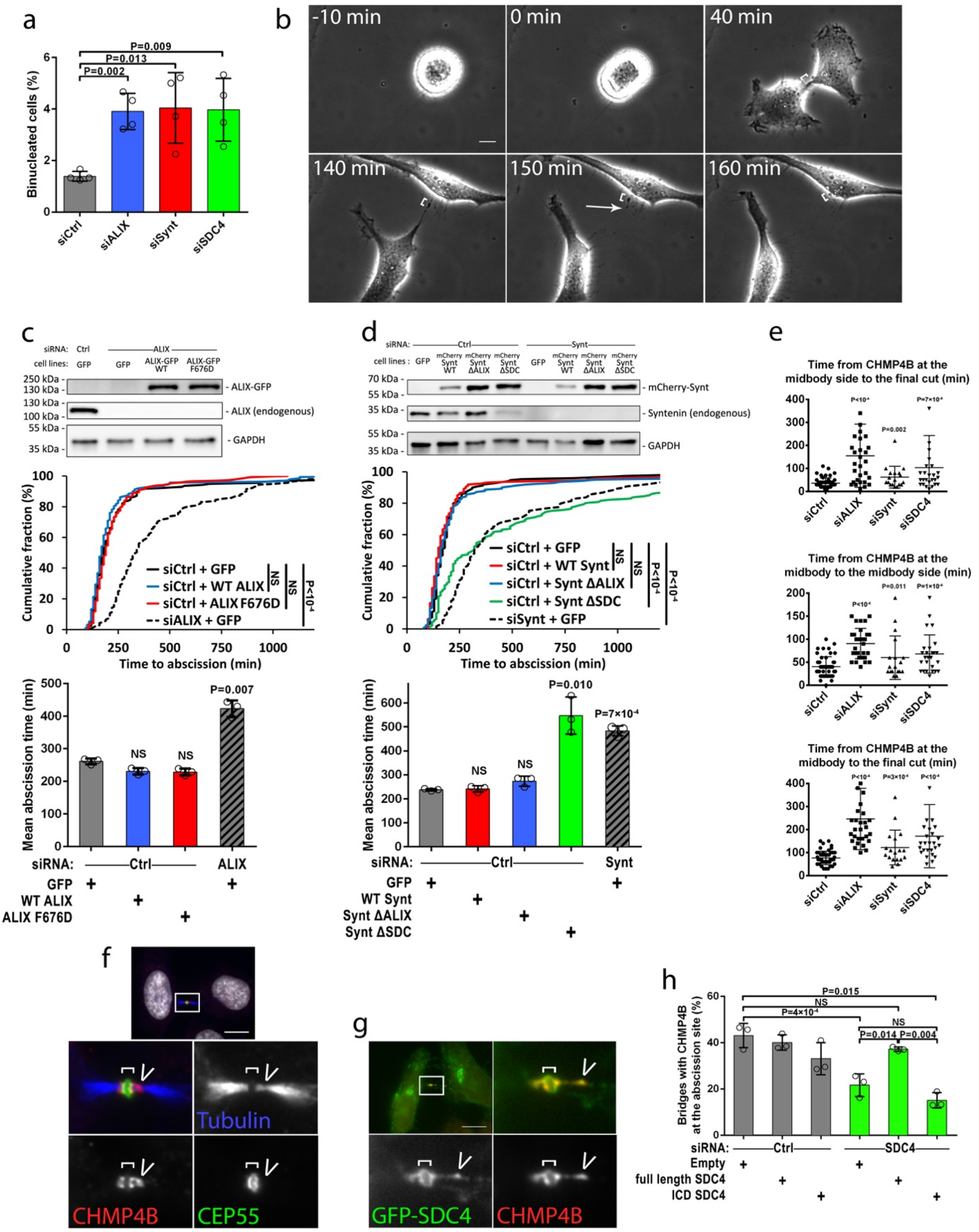

Supplementary Figure 5

Supplementary Fig. 5: Additional cytokinetic phenotypes upon ALIX, syntenin and syndecan-4 depletion.

(a) HeLa cells were treated with either control, ALIX, syntenin or syndecan-4 siRNAs. The percentage (mean ± SD) of cells that were binucleated was quantified, as indicated. n=296-616 cells, N=4 independent experiments, one-sided Student's t-tests.

(b) Snapshots of a representative phase-contrast time-lapse microscopy movie. Time 0 corresponds to the time frame preceding furrow ingression. The abscission occurred at 150 min after furrow ingression and is indicated with an arrow. Scale bar : 10 µm.

(c) Upper panel: cells described in Fig. 4a were analyzed by western blots using anti-GFP or anti-ALIX, as indicated. Loading control: GAPDH . Middle and lower panels: Controls that were not included in Fig. 4a (but that were acquired at the same time) are provided. siCtrl + GFP and siALIX + GFP are thus identical as in Fig. 4a and are given for comparison. KS tests for distributions: NS: non significant. Mean abscission times (means ± SD): one-sided Student's t-tests. n=111-156 cells, N=3 independent experiments. NS: non significant.

(d) Upper panel: lysates from cells described in Fig. 4b were analyzed by western blots using anti-mCherry or anti-syntenin, as indicated. loading control :GAPDH . Middle and lower panels: Controls that were not included in Fig. 4b (but that were acquired at the same time) are provided. siCtrl + GFP and siSyntenin + GFP are thus identical as in Fig. 4b and are given for comparison. Note that syntenin ΔSDC has a dominant negative effect. KS tests for distributions: NS: non significant. Mean abscission times (means ± SD): one-sided Student's t-tests, n=105-147 cells, N=3 independent experiments. NS: non significant.

(e) Upper panel: Quantification of the time (min) elapsed from the first appearance of CHMP4B at the side of the midbody to the event of abscission from movies treated as in Fig. 5 f-i. Each dot represents the measurement from a single movie.

Middle panel: quantification of the time (min) elapsed from CHMP4B enrichment at the midbody to the first appearance of CHMP4B at the side of the midbody from movies treated as in Fig5 f-i. Each dot represents the measurement from a single movie.

Lower panel: quantification of the time (min) elapsed from CHMP4B enrichment at the midbody to the event of abscission from movies treated as in Fig5 f-i. Each dot represents the measurement from a single movie. n=18-50 cells from 4 independent experiments, means ± SD, one-sided Student's t-tests.

(f) Localization of endogenous CHMP4B and CEP55 in cytokinetic bridges with abscission site, as indicated. Note that CEP55 is absent from the abscission site, where CHMP4B is localized. Brackets and arrowheads mark the midbody and the abscission site, respectively. Scale bar : 10 µm.

(g) Localization of endogenous CHMP4B in GFP-syndecan-4 stable cell line. The CHMP4B staining indicates that this zoomed region corresponds to a cytokinetic bridge fixed

shortly before abscission. Note the colocalization between CHMP4B and syndecan-4. Bracket and arrowhead mark the midbody and the abscission site, respectively. Scale bar : 10 μm.

(h) HeLa cells were treated with either control or syndecan-4 siRNAs and transfected with empty plasmids, plasmids encoding full length or the intracellular domain (ICD) of syndecan-4 (tail alone, without transmembrane domain). Percentage (means ± SD) of cytokinetic bridges with CHMP4B at the abscission site in each condition is indicated. n=23-68 cells, N=3 independent experiments, one-sided Student's t-tests. NS: non significant.

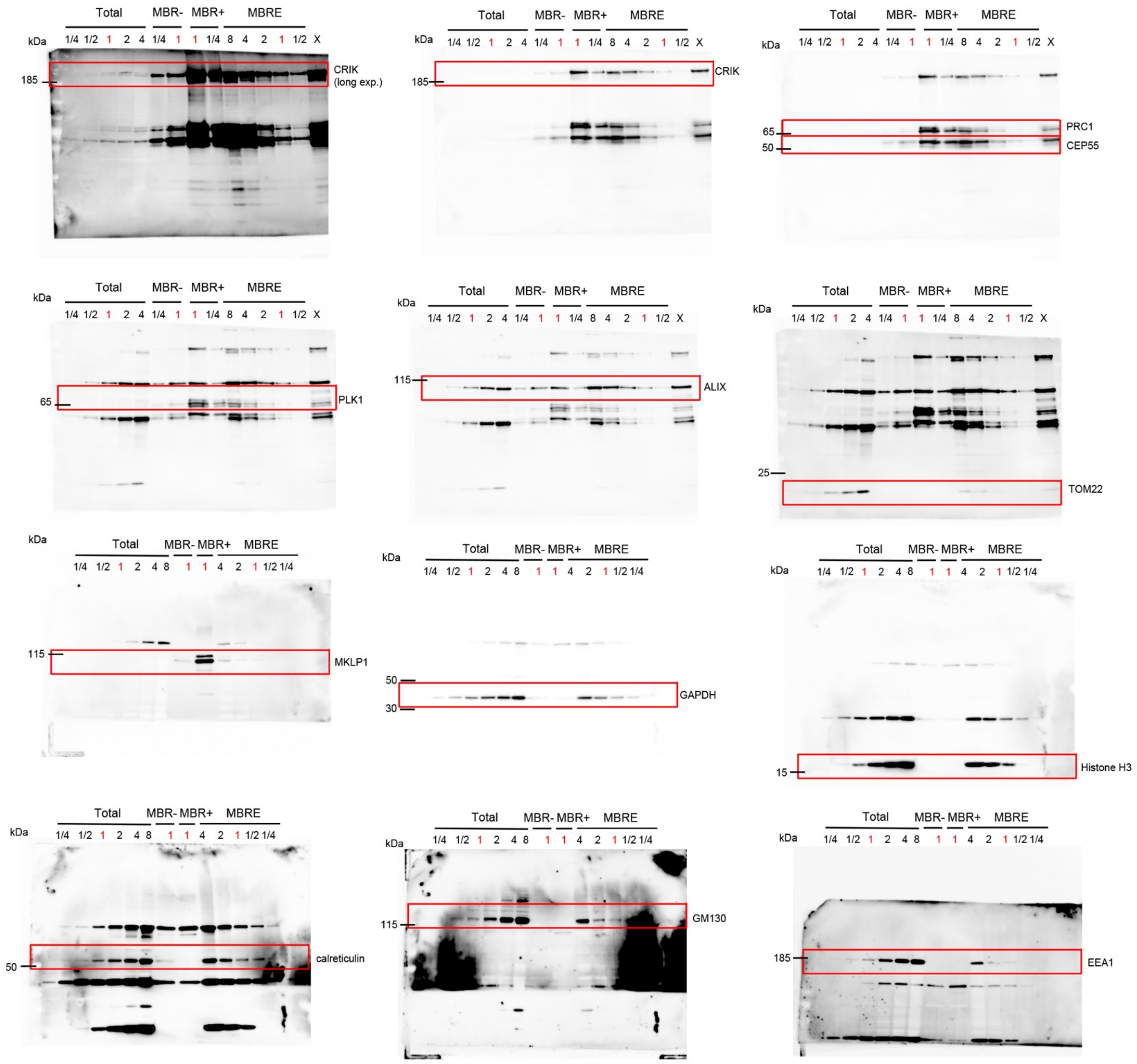

Supplementary Fig. 6

Supplementary Fig. 6: Uncropped western blots displayed in Figure 1.

Membranes were blotted repeatedly with indicated antibodies. Different exposures are provided.

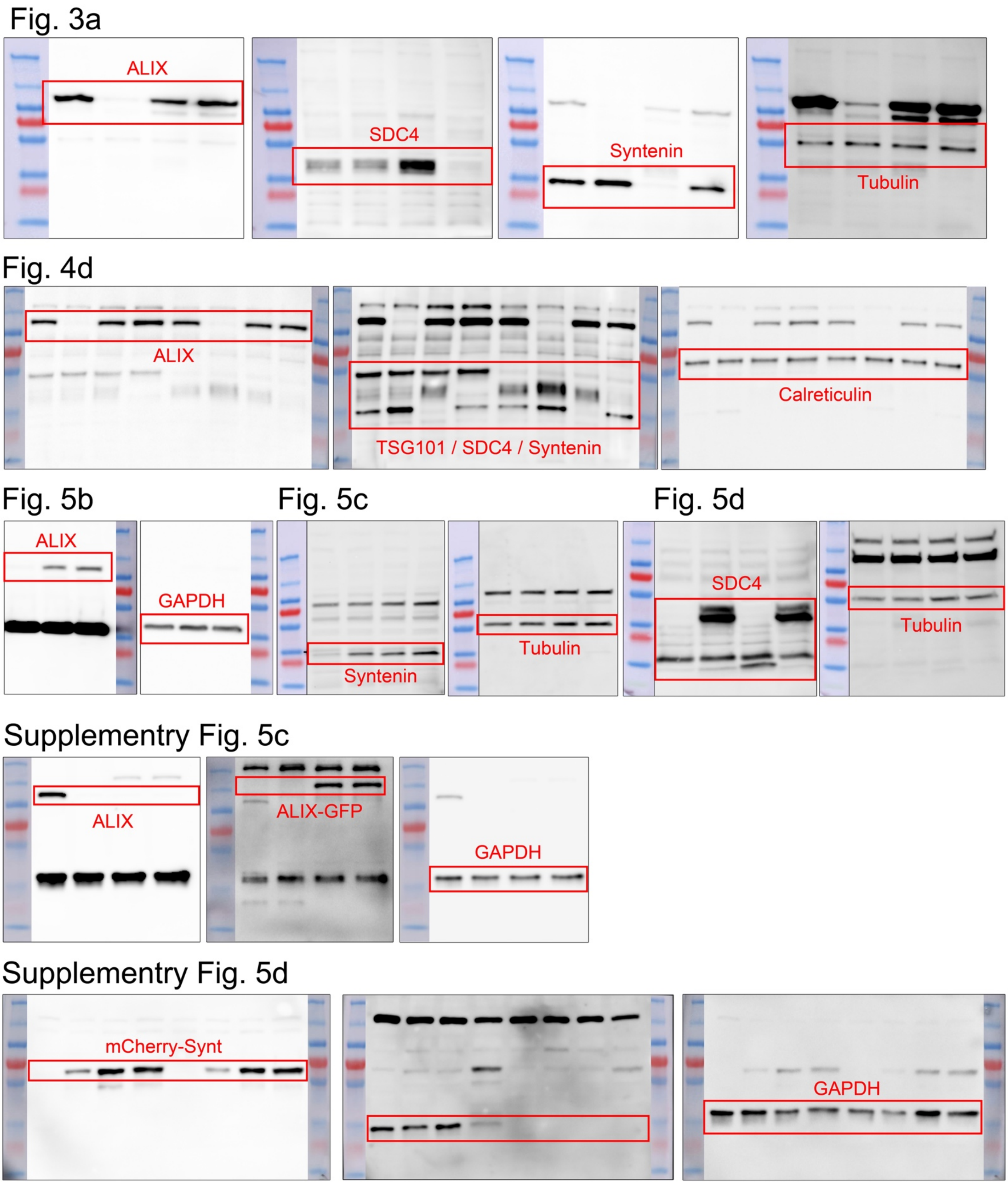

Supplementary Figure 7

Supplementary Fig. 7: Uncropped western blots displayed in Figures 3-5.

Membranes were blotted with indicated antibodies. Different exposures are provided.

Supplementary Table 1: Antibodies, dilutions and fixations used in this study.

All antibodies were raised against Human proteins

target protein	company	host	name/clone	dilution WB	dilution IF	fixation IF
syntenin	homemade	rabbit	Rb2	1/2000	1/500	PFA, MeOH
syntenin	Santa Cruz	mouse	sc-100336 (S-31)		1/500	PFA, MeOH
Alix	Santa Cruz	mouse	sc-271975 (C-11)	1/1000	1/200	MeOH
syndecan-4 ECD	homemade	mouse	8G3		1/2000	PFA
syndecan-4 ICD	AbNova	rabbit	PAB9045	1/1000		
CHMP4B	Proteintech	rabbit	13683-1-AP		1/500	MeOH
tubulin	Sigma	mouse	T4026 (clone TUB 2.1)	1/2000		MeOH
tubulin	Institut Curie	human	F2C-hFc2, VHHD5-hFc1, C3B9-hFc		1/200	PFA, MeOH
GAPDH	Proteintech	mouse	60004-1-Ig (1E6D9)	1/20 000		
TSG101	Proteintech	rabbit	14497-1-AP	1/1000		
Cep55	Santa Cruz	mouse	sc-374051 (B-8)	1/1000	1/500	MeOH,
CRIK	BD Bioscience	mouse	611376	1/2000	1/300	PFA
PLK1	Santa Cruz	mouse	sc-17783 (F-8)	1/1000		
GM130	Abcam	rabbit	EP892Y	1/2000		
TOM22	Sigma	mouse	T6319 (clone 1C9-2)	1/500		
Calreticulin	Abcam	mouse	FMC75 ab22683	1/2000		
MKLP2	homemade	rabbit	Rabkinesin-6		1/2000	PFA
Aurora B	BD biosciences	mouse	BD-611082 (Clone 6)		1/500	MeOH
MKLP1	Santa Cruz	rabbit	sc-867	1/500	1/500	TCA
RacGAP	Abcam	goat	ab2270		1/500	TCA
PRC1	BioLegend	mouse	629001 (6G2)	1/1000	1/500	TCA
Histone H3	Cell Signaling technology	rabbit	9715	1/5000		
EEA1	BD Biosciencce	mouse	14/EEA1	1/500		

MeOH: methanol fixation
PFA: paraformaldehyde fixation
TCA: Trichloracetic acid fixation
