# Supplementary material for: The Flemmingsome reveals an ESCRT-to-membrane coupling via ALIX/syntenin/syndecan-4 required for completion of cytokinesis
Source: Nat Commun. 2020 Apr 22;11:1941. doi: 10.1038/s41467-020-15205-z (PMC7176721; doi:10.1038/s41467-020-15205-z)
Supplement: Supplementary file 3 — Description of Additional Supplementary Information [file 41467_2020_15205_MOESM3_ESM.pdf]

Description of Additional Supplementary Files

File Name: Supplementary Data 1
Description: Proteomic and statistical analysis (Excel file). Supplementary Data provides several tabs: TAB1 Total Flemmingsome: The Flemmingsome or proteome of Midbody Remnants TAB2 Enriched Flemmingsome TAB3 Total proteins identified by mass spectrometry TAB4 Proteins only quantified in MBR+ TAB5 iBAQ values of all the proteins identified by Mass Spectrometry TAB6 Comparative Analysis with the proteome from Skop et al. (Science 2004) TAB7 Clusters of GO terms used in Fig. 1g

File Name: Supplementary Movie 1
Description: ALIX-mScarlet and SiR-tubulin. HeLa cells transiently transfected with a plasmid encoding ALIX-GFP and incubated with SiR-tubulin were recorded by spinning-disk confocal microscopy every 10 min. Time 0 corresponds to the time frame preceding furrow ingression. Scale bar= 10 µm.

File Name: Supplementary Movie 2
Description: Colocalization of CHMP4B-GFP and ALIX-mScarlet at the midbody and at the abscission site. HeLa cells transiently co-transfected with plasmids encoding CHMP4B-GFP and ALIX-mScarlet were recorded by spinning-disk confocal microscopy every 10 min. Merge and individual channels (in grey levels) are provided. Time 0 corresponds to the time frame preceding furrow ingression. Scale bar= 10 µm.

File Name: Supplementary Movie 3
Description: Colocalization of GFP-syntenin and ALIX-mScarlet at the midbody and at the abscission site. HeLa cells transiently co-transfected with plasmids encoding GFP-syntenin and ALIX-mScarlet were recorded by spinning-disk confocal microscopy every 10 min. Merge and individual channels (in grey levels) are provided. Time 0 corresponds to the time frame preceding furrow ingression. Scale bar= 10 µm.

File Name: Supplementary Movie 4
Description: Colocalization of GFP-SDC4 and mScarlet-syntenin at the midbody and at the abscission site. HeLa cells transiently co-transfected with plasmids encoding GFP-SDC4 and mScarlet-syntenin were recorded by spinning-disk confocal microscopy every 10 min. Merge and individual channels (in grey levels) are provided. Time 0 corresponds to the time frame preceding furrow ingression. Scale bar= 10 µm.

File Name: Supplementary Movie 5
Description: CHMP4B-GFP behavior during cytokinesis in control cells. HeLa cells that stably expressed CHMP4B-GFP were treated with control siRNAs and recorded by spinning-disk confocal microscopy every 10 min. Time 0 corresponds to the frame preceding the arrival of CHMP4B at the midbody. The arrow indicates that abscission has occurred.

File Name: Supplementary Movie 6
Description: CHMP4B-GFP behavior during cytokinesis in ALIX depleted cells. HeLa cells that stably expressed CHMP4B-GFP were treated with ALIX siRNAs and recorded by spinning-disk confocal microscopy every 10 min. Time 0 corresponds to the frame preceding the arrival of CHMP4B at the midbody. The arrow indicates that abscission has occurred.

File Name: Supplementary Movie 7
DescriptionCHMP4B-GFP behavior during cytokinesis in syntenin-depleted cells. HeLa cells that stably expressed CHMP4B-GFP were treated with syntenin siRNAs and recorded by spinning-disk confocal microscopy every 10 min. Time 0 corresponds to the frame preceding the arrival of CHMP4B at the midbody. The arrow indicates that abscission has occurred.

File Name: Supplementary Movie 8
Description: CHMP4B-GFP behavior during cytokinesis in syndecan-4-depleted cells. HeLa cells that stably expressed CHMP4B-GFP were treated with syndecan-4 siRNAs and recorded by spinning-disk confocal microscopy every 10 min. Time 0 corresponds to the frame preceding the arrival of CHMP4B at the midbody. The arrow indicates that abscission has occurred.
