# Supplementary material for: The Flemmingsome reveals an ESCRT-to-membrane coupling via ALIX/syntenin/syndecan-4 required for completion of cytokinesis
Source: Nat Commun. 2020 Apr 22;11:1941. doi: 10.1038/s41467-020-15205-z (PMC7176721; doi:10.1038/s41467-020-15205-z)
Supplement: Supplementary file 13 — Reporting Summary [file 41467_2020_15205_MOESM13_ESM.pdf]

nature research

Corresponding author(s): Arnaud ECHARD

Last updated by author(s): Feb 3, 2020

Reporting Summary

Nature Research wishes to improve the reproducibility of the work that we publish. This form provides structure for consistency and transparency in reporting. For further information on Nature Research policies, see Authors & Referees and the Editorial Policy Checklist .

Statistics

For all statistical analyses, confirm that the following items are present in the figure legend, table legend, main text, or Methods section.

n/a	Confirmed	
☐	☒	The exact sample size (*n*) for each experimental group/condition, given as a discrete number and unit of measurement
☒	☐	A statement on whether measurements were taken from distinct samples or whether the same sample was measured repeatedly
☐	☒	The statistical test(s) used AND whether they are one- or two-sided<br>*Only common tests should be described solely by name; describe more complex techniques in the Methods section.*
☒	☐	A description of all covariates tested
☒	☐	A description of any assumptions or corrections, such as tests of normality and adjustment for multiple comparisons
☐	☒	A full description of the statistical parameters including central tendency (e.g. means) or other basic estimates (e.g. regression coefficient) AND variation (e.g. standard deviation) or associated estimates of uncertainty (e.g. confidence intervals)
☐	☒	For null hypothesis testing, the test statistic (e.g. *F*, *t*, *r*) with confidence intervals, effect sizes, degrees of freedom and *P* value noted<br>*Give P values as exact values whenever suitable.*
☒	☐	For Bayesian analysis, information on the choice of priors and Markov chain Monte Carlo settings
☒	☐	For hierarchical and complex designs, identification of the appropriate level for tests and full reporting of outcomes
☒	☐	Estimates of effect sizes (e.g. Cohen's *d*, Pearson's *r*), indicating how they were calculated

Our web collection on statistics for biologists contains articles on many of the points above.

Software and code

Policy information about availability of computer code

Data collection	Microscopy data collection with MetaMorph software (version 7.8.0.0).
Data analysis	Adobe Photoshop CS4 (version 11.0) was used for making Figures. GraphPad Prism (version 6.0c) was used for statistics. For protein identification all data were searched using Andromeda against a Human Uniprot database (downloaded in 20150818, 20204 entries). Functional association network was displayed using Cytoscape (version 3.7.0).

For manuscripts utilizing custom algorithms or software that are central to the research but not yet described in published literature, software must be made available to editors/reviewers. We strongly encourage code deposition in a community repository (e.g. GitHub). See the Nature Research guidelines for submitting code & software for further information.

Data

Policy information about availability of data

All manuscripts must include a data availability statement. This statement should provide the following information, where applicable:

- Accession codes, unique identifiers, or web links for publicly available datasets
- A list of figures that have associated raw data
- A description of any restrictions on data availability

The mass spectrometry proteomics data have been deposited to the ProteomeXchange Consortium via the PRIDE partner repository with the dataset identifier PXD013219.
The Flemmingsome Website: https://flemmingsome.pasteur.cloud/
All material described in this paper will be made available to readers and be sent upon request.

Field-specific reporting

Please select the one below that is the best fit for your research. If you are not sure, read the appropriate sections before making your selection.

[x] Life sciences [ ] Behavioural & social sciences [ ] Ecological, evolutionary & environmental sciences

For a reference copy of the document with all sections, see nature.com/documents/nr-reporting-summary-flat.pdf

Life sciences study design

All studies must disclose on these points even when the disclosure is negative.

Sample size	No statistical method was used to predetermine sample size.
Data exclusions	No data exclusion.
Replication	Reproducibility was confirmed. The number of experiments is described in each Figure legend (N >= 3 independent experiments)
Randomization	We assessed cells from multiple fields for each experiment. The fields were chosen randomly. Once a field was determined, we counted all cells which match the criteria within the field.The number of cells in each experiment is indicated in the relevant Figure Legends.
Blinding	The investigators were not blinded to the sample ID during experiments and outcome assement since cells were counted objectively from randomly selected area.

Reporting for specific materials, systems and methods

We require information from authors about some types of materials, experimental systems and methods used in many studies. Here, indicate whether each material, system or method listed is relevant to your study. If you are not sure if a list item applies to your research, read the appropriate section before selecting a response.

Materials & experimental systems

n/a	Involved in the study
[ ]	[x] Antibodies
[ ]	[x] Eukaryotic cell lines
[x]	[ ] Palaeontology
[x]	[ ] Animals and other organisms
[x]	[ ] Human research participants
[x]	[ ] Clinical data

Methods

n/a	Involved in the study
[x]	[ ] ChIP-seq
[ ]	[x] Flow cytometry
[x]	[ ] MRI-based neuroimaging

Antibodies

Antibodies used	The following antibodies were used in this study (Supplementary Table 2): syntenin homemade rabbit (Rb2); Alix SantaCruz mouse sc-271975 (C-11); syndecan-4ECD homemade mouse (8G3); syndecan-4 ICD AbNova rabbit (PAB9045); CHMP4B Proteintech rabbit (13683-1-AP); tubulin Sigma mouse T4026 (cloneTUB 2.1); tubulin InstitutCurie human (F2C-hFc2,VHHD5-hFc1,C3B9-hFc); GAPDH Proteintech mouse 60004-1-Ig (1E6D9); Cep55 SantaCruz mouse (sc-374051, B-8); CRIK BDBioscience mouse (611376); PLK1 SantaCruz mouse (sc-17783 F-8); GM130 Abcam rabbit (EP892Y); TOM22 Sigma mouse (T6319 clone1C9-2); Calreticulin Abcam mouse (FMC75 ab22683); MKLP2 homemade rabbit Rabkinesin-6; AuroraB BDbiosciences mouse (BD-611082); MKLP1 SantaCruz rabbit (sc-867); RacGAP Abcam Goat (ab2270); PRC1 BioLegend mouse (629001 6G2); HistoneH3 CellSignaling technology rabbit (9715); EEA1 BDBiosciencce mouse (14/EEA1)
Validation	Syntenin, Alix, Syndecan-4 ECD/ICD antibodies were validated by siRNA experiments in this study using westernblots and immunofluorecence (figure 3a, 4d, 5b, 5c, 5d and S4c). The other antibodies have been validated in our previous studies (see PMID 21706022, PMID 28230050).

Eukaryotic cell lines

Policy information about cell lines

Cell line source(s)	HeLa cells were from American Type Culture Collection (ATCC) Clone CCL2. Other cell lines were derived from this parental cell line.
Authentication	Cell lines were authenticated by RT PCR and sequencing.

Mycoplasma contamination	We confirm that all cell lines were tested negative for mycoplasma contamination.
Commonly misidentified lines (See ICLAC register)	No commonly mis-identified cell lines were used in this study.

Flow Cytometry

Plots

Confirm that:

- [x] The axis labels state the marker and fluorochrome used (e.g. CD4-FITC).
- [x] The axis scales are clearly visible. Include numbers along axes only for bottom left plot of group (a 'group' is an analysis of identical markers).
- [x] All plots are contour plots with outliers or pseudocolor plots.
- [x] A numerical value for number of cells or percentage (with statistics) is provided.

Methodology

Sample preparation	MBRs were detached from HeLa GFP-MKLP2 cells with EDTA-treatment as described above. The supernatant from the first 70 g centrifugation was collected. Sorting of MBR+ and MBR- particles was performed on a BD Biosciences FACS ARIA III. Neutral Density filter 1.0 has been used to detect small particles. 65 000 particles were gated on a pseudo-color plot looking at GFP versus SSC-A parameters, both in log scales.
Instrument	BD FACSAriaIII
Software	BD FACSDiva was used to analyze the Flow Cytometry Data (version 10.0.8).
Cell population abundance	N/A (midbody sorting)
Gating strategy	Cells have been excluded from the sorting gates after analysis of an unstained cell suspension as control (see gates in Fig. 1b and Supplementary Fig. 1b). The low SSC populations containing MBR+ (GFP-positive population) and MBR- (GFP-negative counterpart) populations were sorted.

- [x] Tick this box to confirm that a figure exemplifying the gating strategy is provided in the Supplementary Information.
